# Structural influence on titanium ion dissolution in 3D-printed Ti6Al4V orthopedic implants

**DOI:** 10.1038/s41598-025-21129-9

**Published:** 2025-10-23

**Authors:** Eunhyeok Seo, Yu Na Lee, Woo Yeong Shin, Kyung-Hwan Kim, Se Hoon Jung, Hyun Guy Kang, Ryeohyun Kim, Hyokyung Sung, Im Doo Jung, Jong Woong Park

**Affiliations:** 1 Department of Mechanical Engineering, Ulsan National Institute of Science and Technology, UNIST-Gil 50, Ulsan, 44919 Republic of Korea; 2 Surgical Oncology Branch, Division of Clinical Research, National Cancer Center, Goyang, 10408 Republic of Korea; 3 Analysis and Assessment Group, Research Institute of Industrial Science and Technology, Pohang, 37673 Republic of Korea; 4 Center for Rare Cancers, Orthopaedic Oncology Clinic, National Cancer Center, Goyang, 10408 Republic of Korea; 5 Department of Materials Science and Engineering, Kookmin University, Seoul, 02707 Republic of Korea

**Keywords:** Orthopedic implants, Additive manufacturing, 3D-printed Ti6Al4V, Microstructure, Dissolution, Medical research, Engineering, Materials science

## Abstract

3D printed orthopedic implants have emerged as innovative solutions for treating bone tumors, offering advantages such as patient-specific customization and faster production compared to conventional manufacturing methods. However, elevated concentrations of titanium (Ti) ions in the bloodstream have frequently been observed following limb salvage surgery using 3D printed Ti6Al4V implants, which could lead to systemic toxicity and critical implant failure. In this study, we characterize the Ti dissolution phenomenon associated with 3D printed implants. Finite element analysis (FEA) of full-scale pelvic and tibial implants revealed that large mesh surface areas designed for implant–tissue integration can accelerate corrosion. Microstructural analyses of cubical Ti6Al4V samples with solid, mesh, and solid-mesh hybrid geometries revealed that galvanic coupling between the alpha (α) and beta (β) phases drives localized corrosion. A notable difference in β-phase content—ranging from 145% to 200%—was observed among the three cases, with the highest β-phase content in the mesh structures. These findings indicate that although mesh structures are essential for implant–tissue bonding, they can significantly promote Ti ion release, potentially compromising the mechanical integrity of the implant over time. Careful design and surface treatment strategies are therefore needed to balance biological integration with long-term material stability.

## Introduction

Additive manufacturing (AM) has been widely used in various industries, including automobiles, aerospace, smart factories, and the medical industry, enabling on-demand production with high design flexibility^1–6^. AM, notably in the medical field, can utilize various materials such as polymers, ceramics, and metals, providing a range of mechanical properties suitable for internal soft tissues and hard tissues^7–12^. Ti-6Al-4 V alloy, a material frequently used in AM processes, has been used in the production of dental implants and orthopedic implants due to its excellent mechanical properties, biocompatibility, passivity, and corrosion resistance^13–18^. Bone tumors compromise bone strength and adversely affects weight distribution and muscle support^19,20^. These aspects can substantially impact the quality of life of patient by impeding normal mobility and daily activities^21,22^. Successful recovery requires swift removal of the minimally affected bone area, followed by immediate replacement with a biocompatible metal implant within a two-week timeframe^23–26^. In this context, laser powder bed fusion (L-PBF), a type of AM process, has emerged as a popular strategy for fabricating implants characterized by high mechanical performance, fast production, and complex structures^27–29^.

The use of 3D-printed implants for bone reconstruction in bone tumor surgery is becoming increasingly prevalent^30–33^. Although the whole-blood concentration has been reported to increase after using 3D-printed implants, the underlying mechanism remains inadequately explored. This study hypothesizes that the release of metal debris, such as sintered unmelted powder, from the alloy surface treated through powder bed fusion may constitute the main mechanism. This phenomenon may be attributable to the large surface area resulting from elevated surface roughness, even in solid structures, or the presence of special structures such as lattices.

Ti alloys have been widely used for fabricating orthopedic implants^34–39^, and an increase in the concentration of metals present in the alloy, including Ti, has not been observed to pose toxicity risks to the human body^40,41^. However, a significant elevation in Ti concentration can serve as a surrogate marker for potential mechanical failure,^42–45^ such as breakage or cracking of the implant. Additionally, the concentrations of Al and V, typically used to enhance mechanical performance of implants, may also increase, posing a potential toxicity risk^46–49^.

In addition to the toxicological aspects associated with the increase in concentrations of Ti, Al, and V, if the rise in whole-blood concentration is primarily attributed to corrosion, a detailed analysis of this mechanism is essential. Specifically, it is crucial to ascertain whether the accelerated corrosion is attributable to increased surface area owing to the use of a porous structure, such as lattices, or if corrosion is induced at the interface between solid and lattice structures. If the increase in surface area is the determining factor, even with an initially high concentration, metal dissolution may reach a plateau once bone and soft tissue adhesion has sufficiently progressed. Investigating whether this blood concentration poses toxicological problems becomes paramount. However, if corrosion is accelerated at the solid–lattice interface, further attention may be warranted, as this phenomenon has the potential to initiate cracks or degrade mechanical performance. To address such complex mechanisms, computational approaches have also been increasingly employed to investigate corrosion processes in metallic biomaterials. COMSOL Multiphysics, in particular, has been widely used to simulate electrochemical corrosion phenomena such as localized corrosion, galvanic interactions, and ion transport in titanium alloys. Recent work by Sharma et al. employed COMSOL to model the corrosion behavior of additively manufactured iron structures in physiological electrolytic environments, and numerous other studies have also utilized COMSOL for simulating corrosion processes in metallic materials, indicating its broad application for such analyses^50–52^.

Therefore, the objective of this study was to identify the causes underlying the increase in blood Ti concentration after surgery involving a 3D-printed bone replacement implant. To this end, we conducted a quantitative comparison to determine whether the increase in blood Ti levels is attributable to metal debris released from the alloy surface or corrosion. Additionally, we analyzed variations in the metal concentration with the specimen structure and surface area. Furthermore, we investigated whether the elution amount in the 3D-printed specimen exceeds that of solid and form cell porous (lattice) structures produced using conventional manufacturing methods. Quantitative results were obtained through computer analysis and polarization experiments, designed to identify any mechanically weakened areas owing to local concentrations of metal elution.

## Methods and materials

### Materials and fabricating specimens

Three types of specimens were designed for the experiment. All specimens were cubes with a side length of 20 mm, characterized by full-solid structures, full-lattice structures, and hybrid structures with solid and lattice components (lattice unit size: 2 mm). L-PBF was selected as the 3D printing method, with 20 specimens of each structure printed using Ti6Al4V ELI with a particle size between 15 and 53 μm (extra-low interstitial, with higher purity than Ti6Al4V). All 60 specimens (3 structures × 20 specimens of each structure) underwent post-processing, including heat treatment and sandblasting, following the same procedures as those applied to 3D-printed human implants used in actual surgeries. Samples were heated to 900 °C at a rate of 5 °C/min, held for 1 h, and subsequently cooled for 24 h. For sandblasting, particles ranging from 0.125 to 0.250 mm were used, with a blasting pressure of 5 kgf/cm² and a perpendicular (90°) angle of incidence to the target surface. Each blasting cycle lasted within 10 s, with a total processing time not exceeding 30 min. The composition of Ti6Al4V ELI is outlined in Table 1. The chemical composition listed in Table 1 corresponds to the manufacturer’s specification for Ti6Al4V ELI powder, and no additional post-printing analysis was performed.

Corrosion test specimens are fabricated by L-PBF process using Dpert M200 (DAEGUNTECH, Changwon, South Korea). The processing parameters are as follows: laser power of 175 W, laser scan velocity 1050 mm/s, layer thickness of 0.03 mm. The laser selectively melts the metal powder onto the building platform to conform to the desired shape. After melting, the building platform moves downward along the z-axis, and the powder platform moves upward. Subsequently, a recoater applies a new layer of powder onto the building platform. The laser selectively melts the recoated powder, and this process is repeated to attain a 3D model. A commercial implant (MUTARS, Implantcast, Germany) was utilized for the comparison of dissolution phenomenon in ultrapure water and Hartmann’s solution.

**Table 1:** Composition of Ti6Al4V ELI.

Element	Al	V	Fe	O	C	*N*	Ti
Mass (%)	6.5	4.5	0.25	0.13	0.08	0.05	Balance

### Metal concentration measurement

Sets of 10 specimens were submerged in an ultrapure water solution for 7 d. Subsequently, the same specimens were thoroughly washed and submerged for an additional 7 d. Different sets of 10 specimens were submerged in Hartmann’s solution for 7 d. The concentrations of metals in the ultrapure water maintained for 7 d and those in the solution maintained for an additional 7 d after wash-out were compared to determine the wash-out effect. Additionally, the concentrations of metals in the ultrapure water after 7 d were compared with the concentrations in Hartmann’s solution after 7 d to evaluate the corrosive effect. Samples were eluted at room temperature by adding 100 mL each of deionized (ultrapure) water and Hartmann’s solution to a 250 mL polytetrafluoroethylene container with a cap. Subsequently, 14.25 mL of the eluted solution was placed in a 15 mL perfluoroalkoxy (PFA) tube, and 0.75 mL of nitric acid (HNO_3_; distilled grade, TAMAPURE-AA-100, TAMA chemicals, Kawasaki, Japan) was added until the marked line. Hartmann’s solution was diluted 10 times using deionized water. High-resolution inductively coupled plasma mass spectrometry (HR-ICP-MS; ElementXR, Thermo Fischer Scientific, Dreieich, Germany) was used for trace elemental analysis at medium resolution (*R* = 4,000). The operating parameters for ICP-MS are presented in Table 2. The concentrations of the three elements (Ti, V, and Al) in the samples were quantified through external calibration using certified reference material solutions (AccuStandard, New Haven, CT, USA). The chemical composition of Hartmann’s solution is outlined in Table 3.

**Table 2:** Operating conditions for ICP-MS.

ElementXR ICP-MS	Parameter
Plasma forward power (W)	1,250
Argon flow rate (L/min)	-
Plasma	16
Auxiliary	0.8
Nebulizer	1
Sample introduction system	-
Nebulizer	PFA micronebulizer
Spray chamber	PFA Scott type
Carrier flow rate (mL/min)	1.08
Mass resolution	Medium: 4,000
Isotope	^27^Al, ^47^Ti, ^51^V

**Table 3:** Composition of hartmann’s solution.

Compound	CaCl_2_	KCl	NaCl	NaC_3_H_5_O_3_
Concentration (g/L)	0.2	0.3	6	3.1

### Finite element analysis

The FEA was conducted using commercial software (COMSOL Multiphysics v6.0, https://www.comsol.com/) to compare the Ti dissolution results based on the structure of the specimens. The material of the specimen is a Ti6Al4V alloy manufactured by the L-PBF process and then annealed. The simulations were conducted assuming no defects or residual powder remaining. To reduce computational time, 2D cross-sections of each specimen were extracted, and corrosion conditions were applied at the contact region between the specimen and solution. Solid, hybrid, and lattice specimens were modeled using 507, 3457, and 9695 free triangular meshes, respectively. The experimental setup also included simulating the Ti dissolution behavior by immersing the three types of specimens in ultrapure water and Hartmann’s solution for 7 days, replicating the actual test conditions. In addition to the specimen simulations, the FEA was extended to a full pelvic implant model to evaluate the dissolution behavior in a more clinically relevant scenario. The pelvic implant, also made of Ti6Al4V alloy, was modeled to simulate dissolution behavior under conditions replicating those of the human body with Hartmann’s solution as the environment. The 3D model of the implant included both solid and lattice regions, and the dissolution behavior was analyzed through cross-sectional slices at 5 mm intervals. The boundary conditions and simulation parameters were set to be the same as those used for the specimens, targeting the dissolution behavior after 7 d, allowing for a direct comparison between the simple specimens and the more complex implant structure. Parameters for the dissolution simulation, i.e., the diffusion coefficients (D), molar masses (M), and charge numbers of ions (n) are outlined in Table 4. The pH of Hartmann’s solution in the dissolution simulation was set to 7.

Although the dissolution process occurs in three dimensions in vivo, the FEA in this study was conducted using two-dimensional cross-sectional slices extracted from the 3D implant geometry. This approach was chosen to reduce computational complexity while maintaining analytical relevance. Each 2D slice was used to evaluate the localized dissolution behavior of solid and lattice regions under uniform boundary and environmental conditions. Because solid or lattice surfaces do not allow dissolution to occur perpendicular to the plane outside the boundary layer, the simulation results based on 2D sections are sufficient to represent real-world dissolution tendencies in those regions. Therefore, this method enables meaningful comparison of corrosion susceptibility across anatomical regions and provides insight into how implant design influences localized Ti ion release patterns within a clinically relevant 3D structure.

**Table 4:** Dissolution simulation parameters.

Species	D (m^2^/s)	M (g/mol)	*n*
Ti^4+^	9.85e^− 16^	47.867	+ 4
Al^3+^	5.41e^− 10^	26.982	+ 3
V^5+^	7.65e-^14^	50.942	+ 5
OH^−^	5.30e^− 9^	17.01	-1
H^+^	9.30e^− 9^	1.01	+ 1
Ca^2+^	0.79e^− 9^	40.08	+ 2
K^+^	1.96e^− 9^	39.10	+ 1
Na^+^	1.33e^− 9^	22.99	+ 1
Cl^−^	2.03e^− 9^	35.45	-1

## Results

**Fig. 1:**
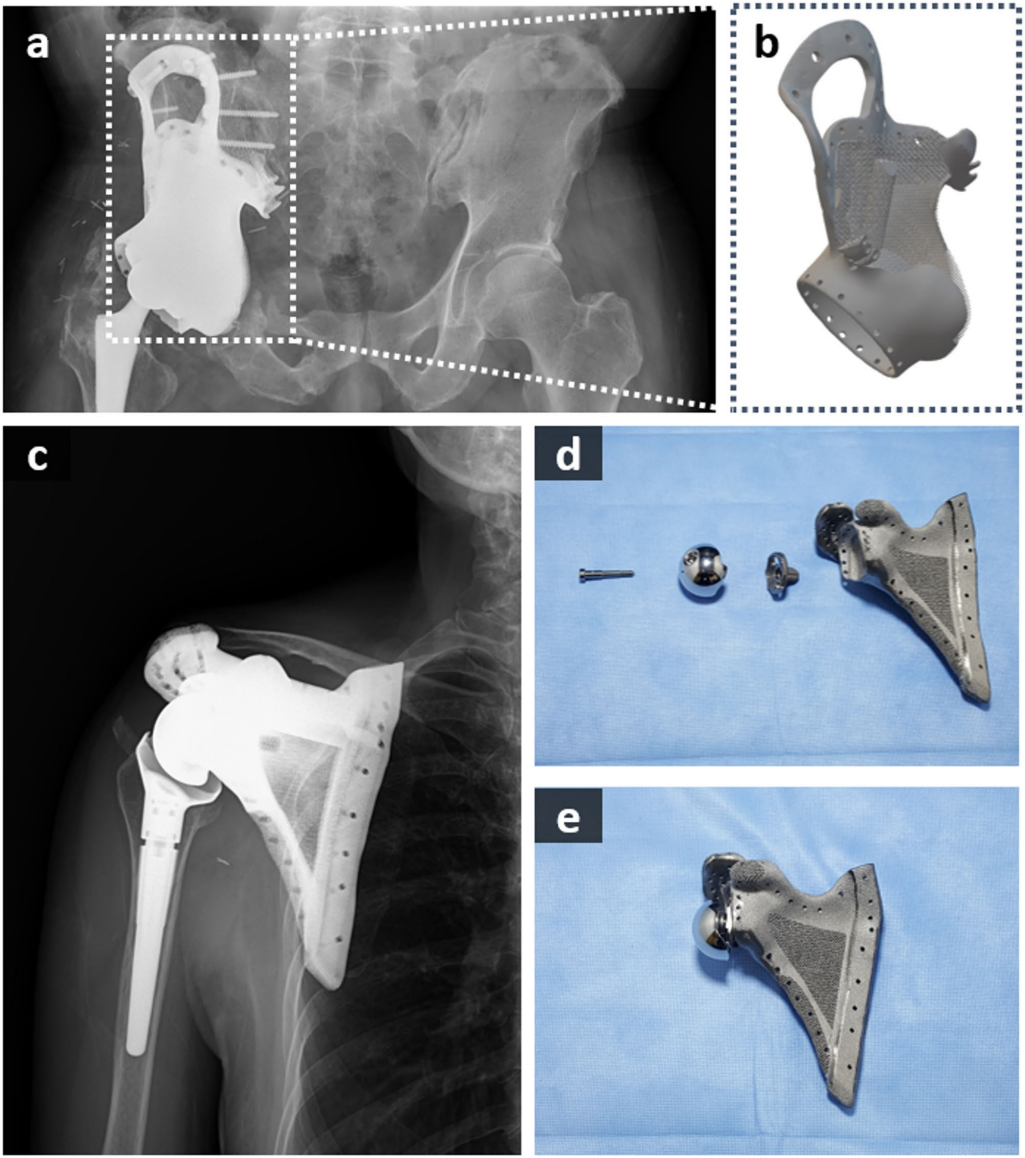
(**a**,** c**) X-ray images of patients after implant surgery (**b**,** d**,** e**) Images of the 3D-printed implants (**b** pelvis, **d-e** scapula) used by the patients.

**Fig. 2:**
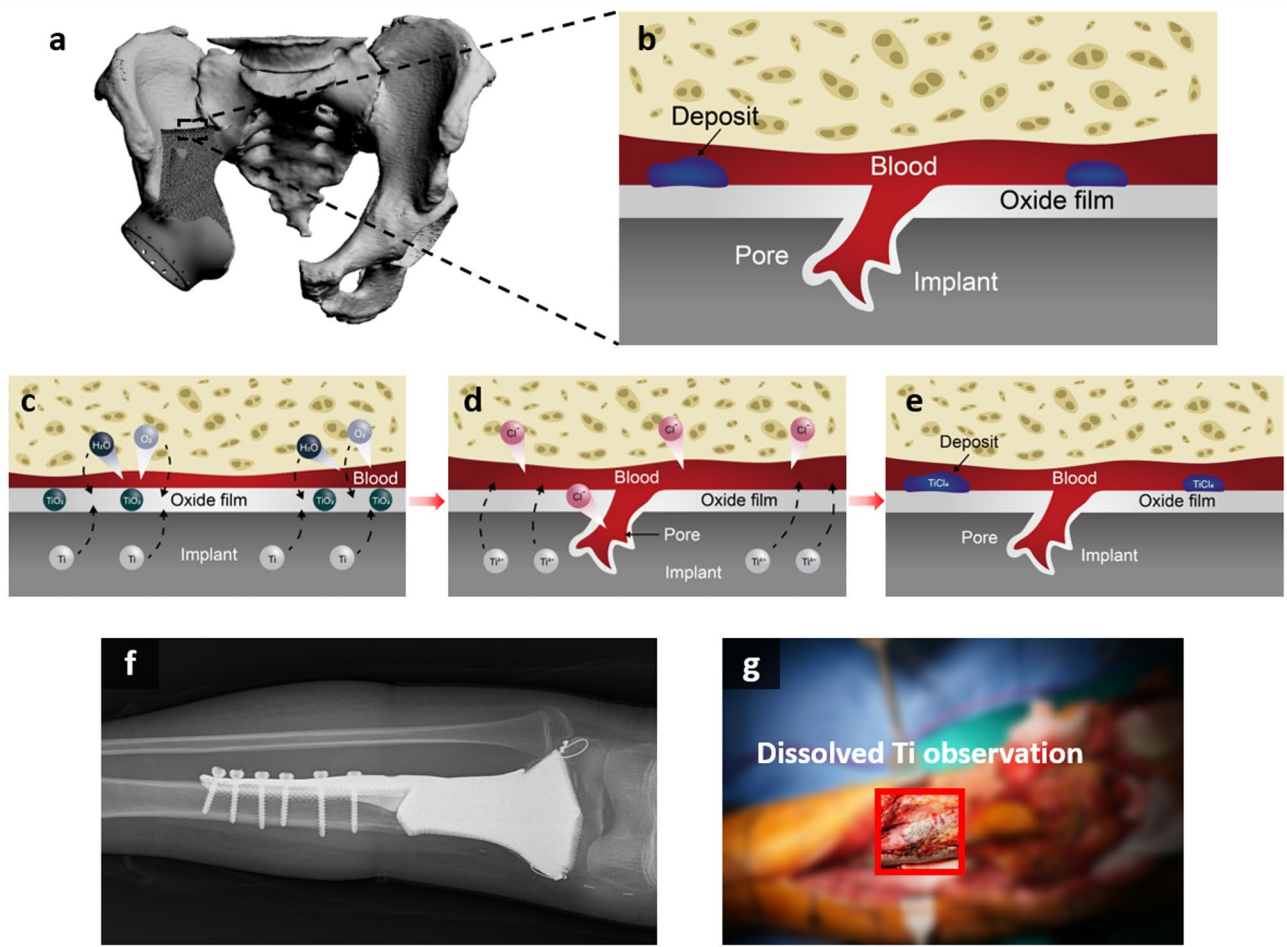
Schematic representation of the Ti dissolution process in a 3D-printed orthopedic implant (**a**) 3D image of the pelvis and implants (**b**) Schematic of the interaction between Ti in the implant and body environment (**c**) Initial interaction between the implant and physiological environment, where the TiO₂ oxide layer forms via reaction with H₂O and O₂ (**d**) Corrosion initiation caused by Cl⁻ ions, leading to the dissolution of Ti⁴⁺ ions through defects in the oxide film. (**e**) Formation of TiCl₄ corrosion products, which inhibit oxide layer regeneration and contribute to Ti ion elution into the bloodstream. Plain radiographs of 3D-printed implant reconstruction for osteosarcoma of the tibia: (**f**) after revision surgery 18 months later due to tumor recurrence. (**g**) Intraoperative photograph showing metal deposition (red box) in the surrounding tissues of the implant.

The X-ray images after limb salvage surgery of an actual patient are shown in Fig. 1a and c. The 3D modeling image consisted of the affected bone of the patient and a 3D-printed orthopedic implant as shown in Figs. 1b and 2a, while Fig. 1d and e show the scapula implant and its assembled components. Metallosis is a complication in which metal is deposited in surrounding soft tissue after metal implantation^53^. This commonly occurs after arthroplasty with metal-on-metal implants in the field of orthopedics. One of the important protective barriers is an oxide layer on the surface of metal implants (Fig. 2c). In situations using as-built 3D-printed metal implant with rough or porous surfaces, metallosis can be accelerated without friction between metals. In a revisional surgery after 18 months from initial surgery (Fig. 2f), metal deposition was observed in the soft tissue around the 3D implant even in areas where there was no friction between metals (Fig. 2g). Corrosion is mainly induced by the interaction of Ti in the implant and Cl^−^ in the blood (Fig. 2d), and Ti ions are eluted from defects in which the oxide film on the implant surface is destroyed (Fig. 2e). During this process, Ti^4+^ and Cl^−^ either generate corrosion deposit or Ti and H_2_O in the blood regenerate the oxide film as schematically shown in Fig. 2b.

**Fig. 3:**
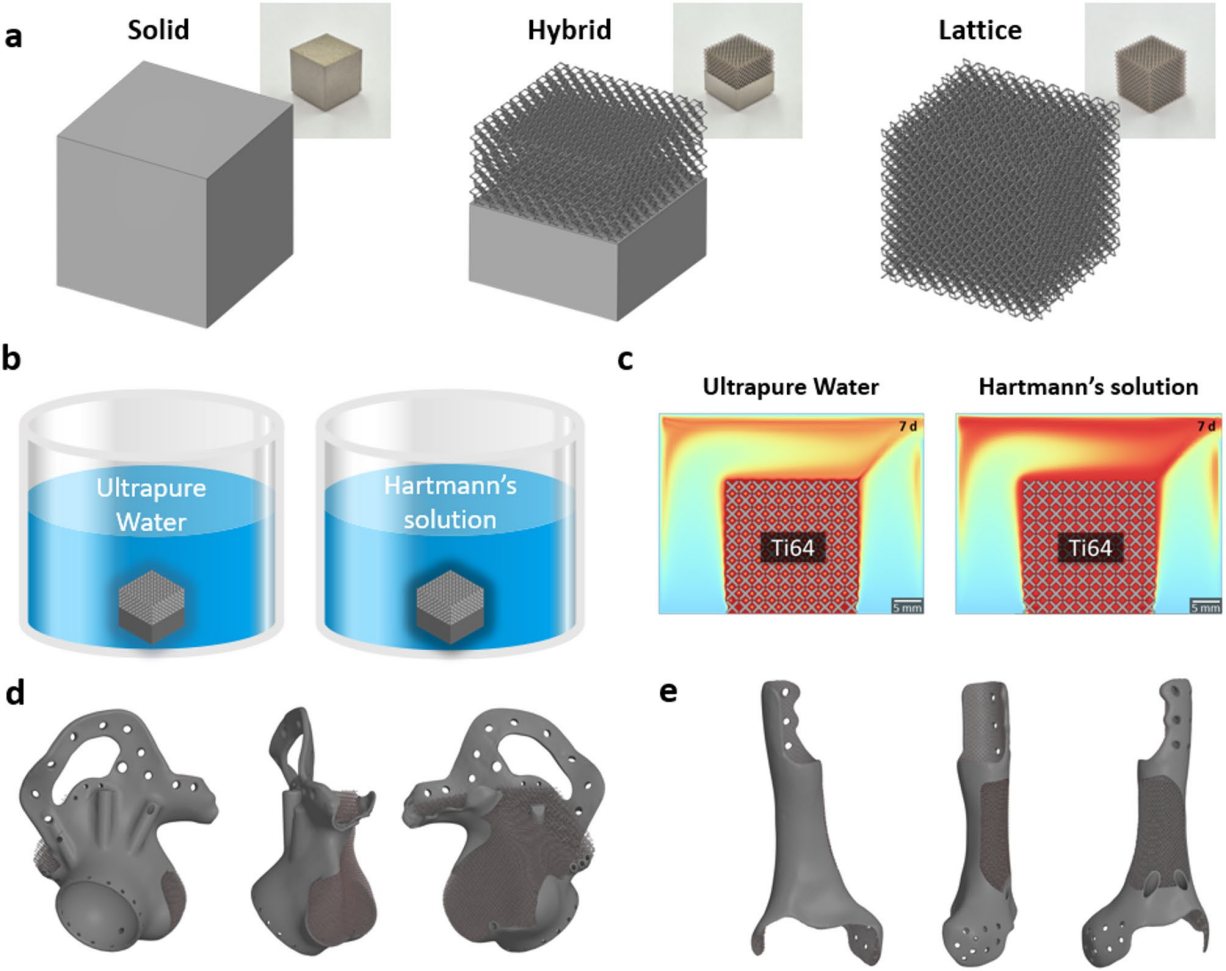
Overview of the dissolution experiments and FEA simulation using three types of 3D-printed specimen. (**a**) Fabricated specimens (modeled using SpaceClaim 2021 R1, https://www.ansys.com/products/3d-design/ansys-spaceclaim) (**b**) dissolution experiment (**c**) FEA simulation (COMSOL Multiphysics v6.0) (**d**) 3D design of the titanium pelvic implant showing solid and lattice regions (modeled using SpaceClaim 2021 R1) (**e**) 3D design of the titanium tibial implant showing solid and lattice regions (modeled using SpaceClaim 2021 R1).

The overview of the dissolution experiments and finite element analysis (FEA) simulation using three types of 3D-printed specimens is shown in Fig. 3. Three types of Ti6Al4V specimens—solid, hybrid, and lattice—were fabricated to assess how structural design influences Ti ion release (Fig. 3a). The inset photographs illustrate the as-built specimens, showing typical surface textures from the additive manufacturing process. These specimens were immersed in ultrapure water and Hartmann’s solution (Fig. 3b), the latter chosen to simulate physiological ionic conditions relevant to the in vivo environment. The 7-day dissolution behavior was analyzed using FEA (Fig. 3c), which visualized the diffusion profiles of Ti ions around the lattice structure, providing comparative insight into the effect of solution chemistry. In addition, 3D models of patient-specific pelvic and tibial implants were reconstructed based on actual clinical implants (Fig. 3d and e). Lattice structures were incorporated into these models to simulate realistic implant geometries and evaluate their dissolution behavior under similar fluid conditions.

Ti alloys undergo elution in two situations: The first situation is when an oxide film is created. Ti reacts with H_2_O in the solution to create the oxide film, and the Ti ions that do not participate in the oxide film formation are eluted. In such cases, an extremely small amount of Ti is eluted even in pure water. The reaction of Ti and H_2_O forms TiO_2_, as indicated in Eq. (1).

1 $$\:\:Ti+2{H}_{2}O\:\to\:\:Ti{O}_{2}+4{H}^{+}+4{e}^{-}$$

The second situation is when the oxide film is destroyed by external factors. When a defect occurs on the implant surface due to friction or impact, Ti in the implant reacts with Cl^−^ ions in the body environment before the TiO_2_ layer is created, causing dissolution of Ti ions. Additionally, Ti ions and Cl^−^ combine to create corrosion products^28^. Corrosion products interfere with the formation of an oxide layer on the implant surface and cause more Ti to be eluted. These Ti^4+^ ions react with Cl^−^ ions in Hartmann’s solution to form titanium tetrachloride (TiCl_4_), as indicated in Eq. (2).

2 $$\:{Ti}^{4+}+{4Cl}^{-}\to\:\:Ti{Cl}_{4}$$

**Fig. 4:**
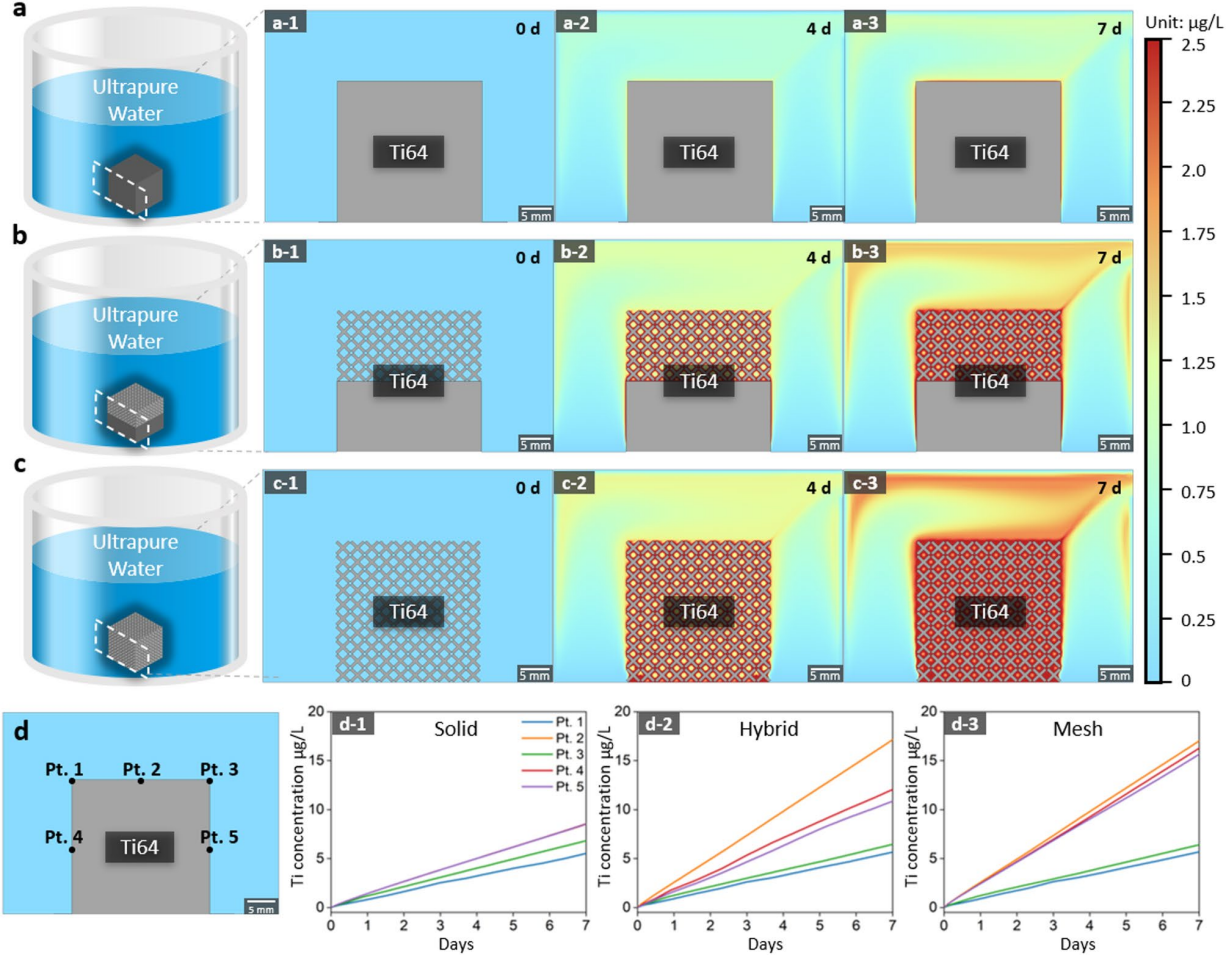
Ti dissolution simulation in ultrapure water: (**a**) solid-structure specimen, (**b**) hybrid-structure specimen, and (**c**) lattice-structure specimen. Variation in Ti concentration over time (**a-1**, **b-1**, **c-1**) before commencing dissolution, (**a-2**, **b-2**, **c-2**) after 4 d, and (**a-3**, **b-3**, **c-3**) after 7 d. (**d**) Visualization of simulated Ti ion concentrations at five specific points (Pt. 1 to Pt. 5) on the structures’ surfaces. (**d-1**) solid, (**d-2**) hybrid, and (**d-3**) mesh structure.

**Fig. 5:**
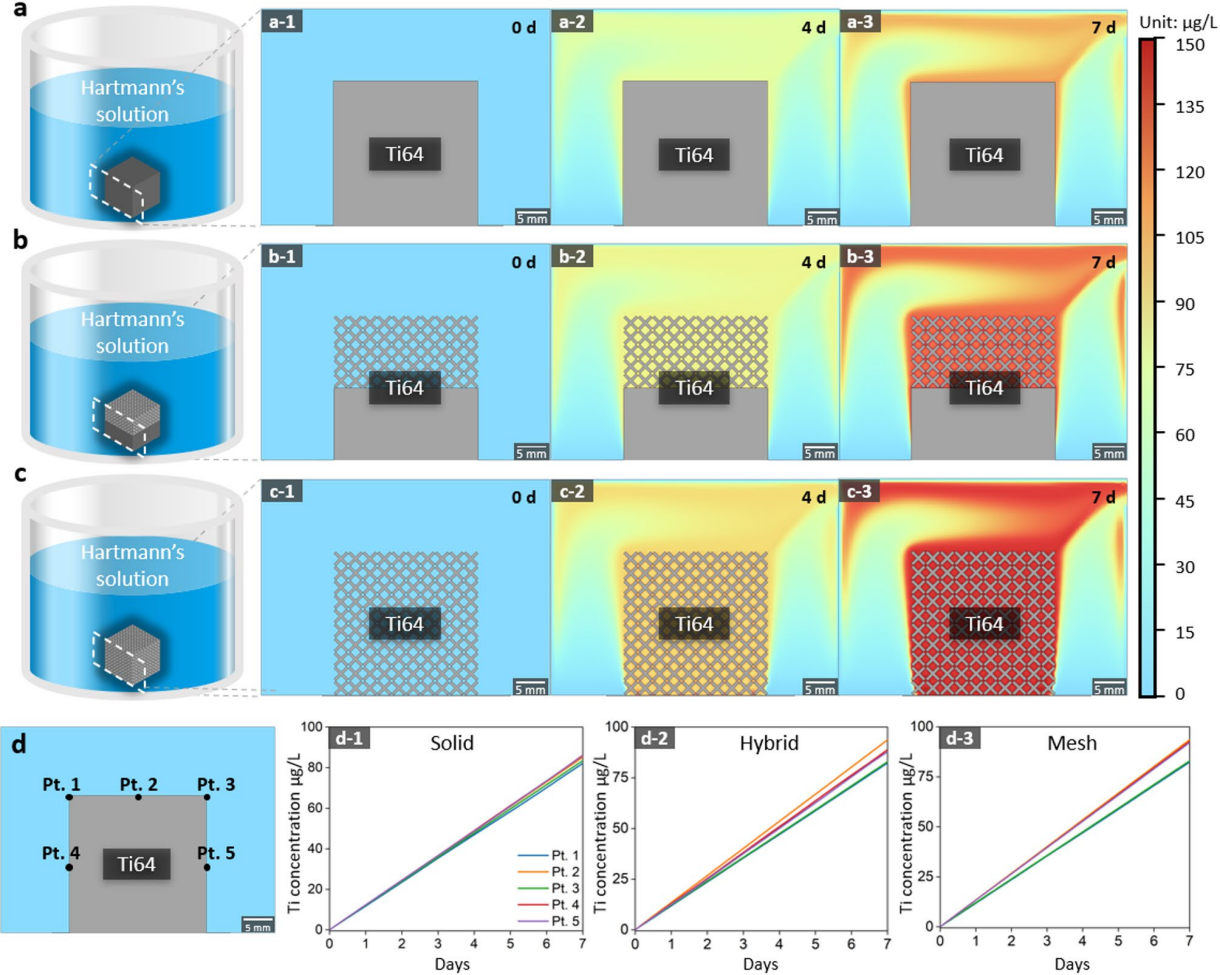
Ti dissolution simulation in Hartmann’s solution: (**a**) solid-structure specimen, (**b**) hybrid-structure specimen, and (**c**) lattice-structure specimen. Variation in Ti concentration over time (**a-1**, **b-1**, **c-1**) before commencing dissolution, (**a-2**, **b-2**, **c-2**) after 4 d, and (**a-3**, **b-3**, **c-3**) after 7 d. (**d**) Visualization of simulated Ti ion concentrations at five specific points (Pt. 1 to Pt. 5) on the structures’ surfaces. (**d-1**) solid, (**d-2**) hybrid, and (**d-3**) mesh structure.

In the FEA simulation of solid, mesh and hybrid structure in ultrapure water, the solid structures exhibited the lowest dissolution amount (concentration of 1.83 µg/L), followed by hybrid (2.04 µg/L) and lattice structures (2.38 µg/L) as Fig. 4. In Hartmann’s solution, the dissolution trend by structure design difference was more distinct as the solid structures exhibiting dissolution amount 82.8 µg/L, hybrid 90.5 µg/L, and lattice structures 128 µg/L as Fig. 5. The Ti concentration increased with the expanding contact surface area between the specimens and solution, with approximately 50 times more dissolution observed in Hartmann’s solution compared with ultrapure water. Additionally, Figs. 4d and 5d illustrate the simulated Ti ion concentrations at five specific points (Pt. 1 to Pt. 5) on the structures’ surfaces, visualized as linear graphs. The trends in Ti ion concentration over time (d-1, d-2, and d-3) correspond to the solid, hybrid, and mesh structures, respectively. The graphs indicate that the dissolution rate increases consistently with the complexity of the structure and the surface area exposed to the solution, with the mesh structure exhibiting the highest dissolution rate at all measured points.

**Fig. 6:**
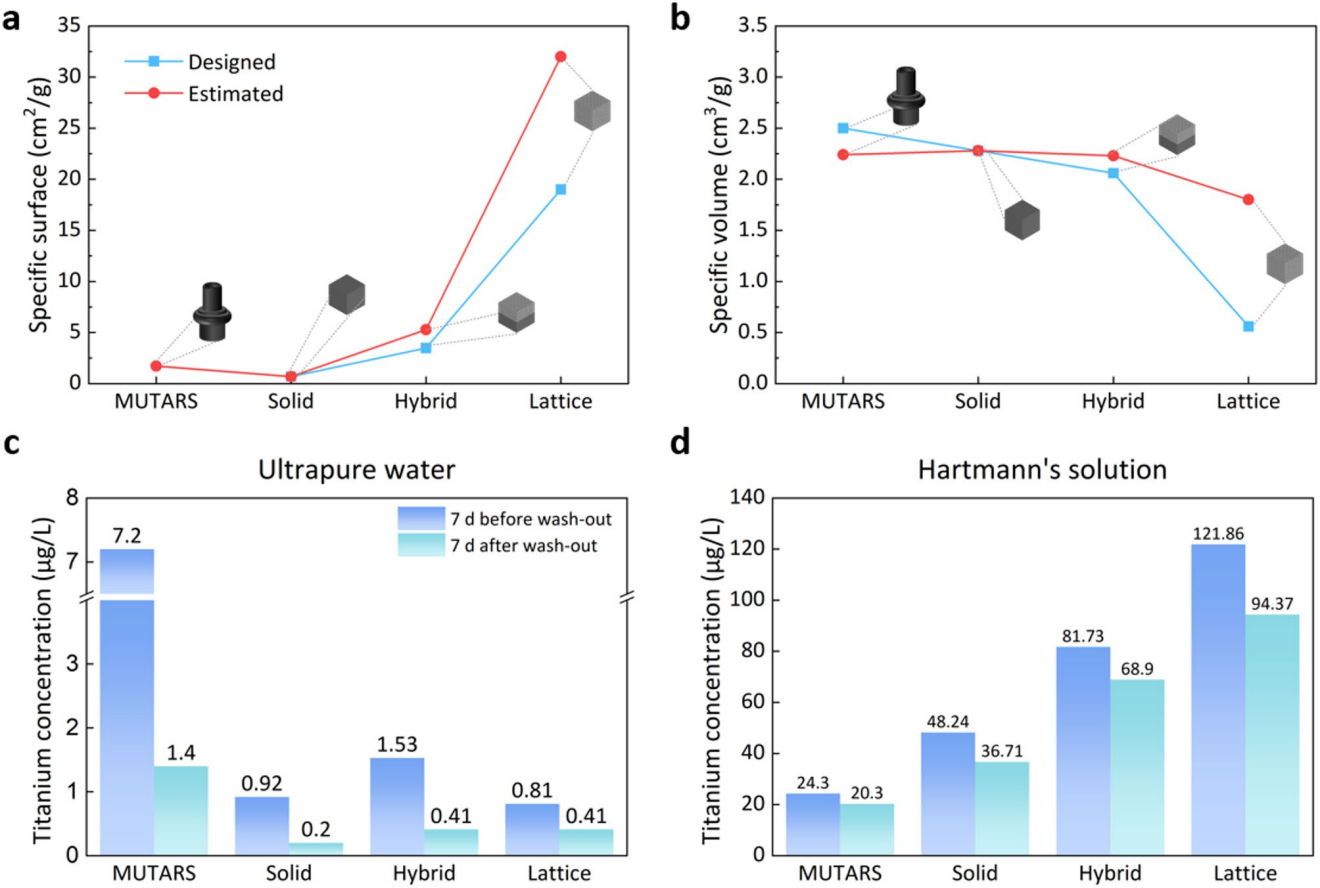
Specific surface area (**a**) and specific volume (**b**) of MUTARS and L-PBF specimens, comparing designed values (blue squares) and estimated values (red circles). Comparison of dissolution experiment results for samples with different structures (solid, hybrid, lattice) and MUTARS (Implantcast) in (**c**) ultrapure water and (**d**) Hartmann’s solution.

The specific surface area and specific volume of MUTARS and L-PBF specimens before dissolution experiments are shown in Fig. 6a and b. Specific surface areas of original 3d design are 0.68, 1.71, 3.48 and 19 cm2/g for solid, hybrid, MUTARS, lattice respectively. However, the hybrid and lattice structure of actual fabricated parts exhibited higher specific surface area as 5.29 and 32 cm2/g respectively. These increases of surface area after L-PBF process can be originated from the existence of partially unmelt powder around lattice structure.

During the initial 7 d in ultrapure water and subsequent 7 d after wash-out, MUTARS exhibited a higher Ti dissolution concentration compared with the 3D-printed solid specimen (Fig. 6c). However, in the experiment with Hartmann’s solution, the 3D-printed solid specimen displayed a higher dissolution concentration than MUTARS (Fig. 6d). Compared with conventionally fabricated specimens, 3D-printed solid specimens exhibit high surface roughness and a larger number of defects^54^. The increased number of defects results in a larger area susceptible to pitting corrosion compared with conventionally fabricated parts along with higher elution of Ti ions.

Before wash-out, no dissolution trend of the Ti6Al4V alloy was observed in ultrapure water and Hartmann’s solution. After wash-out, the trend of the concentration of Ti ions to increase as the specific surface area of the specimen increases was confirmed after the dissolution experiments in both solutions.

**Fig. 7:**
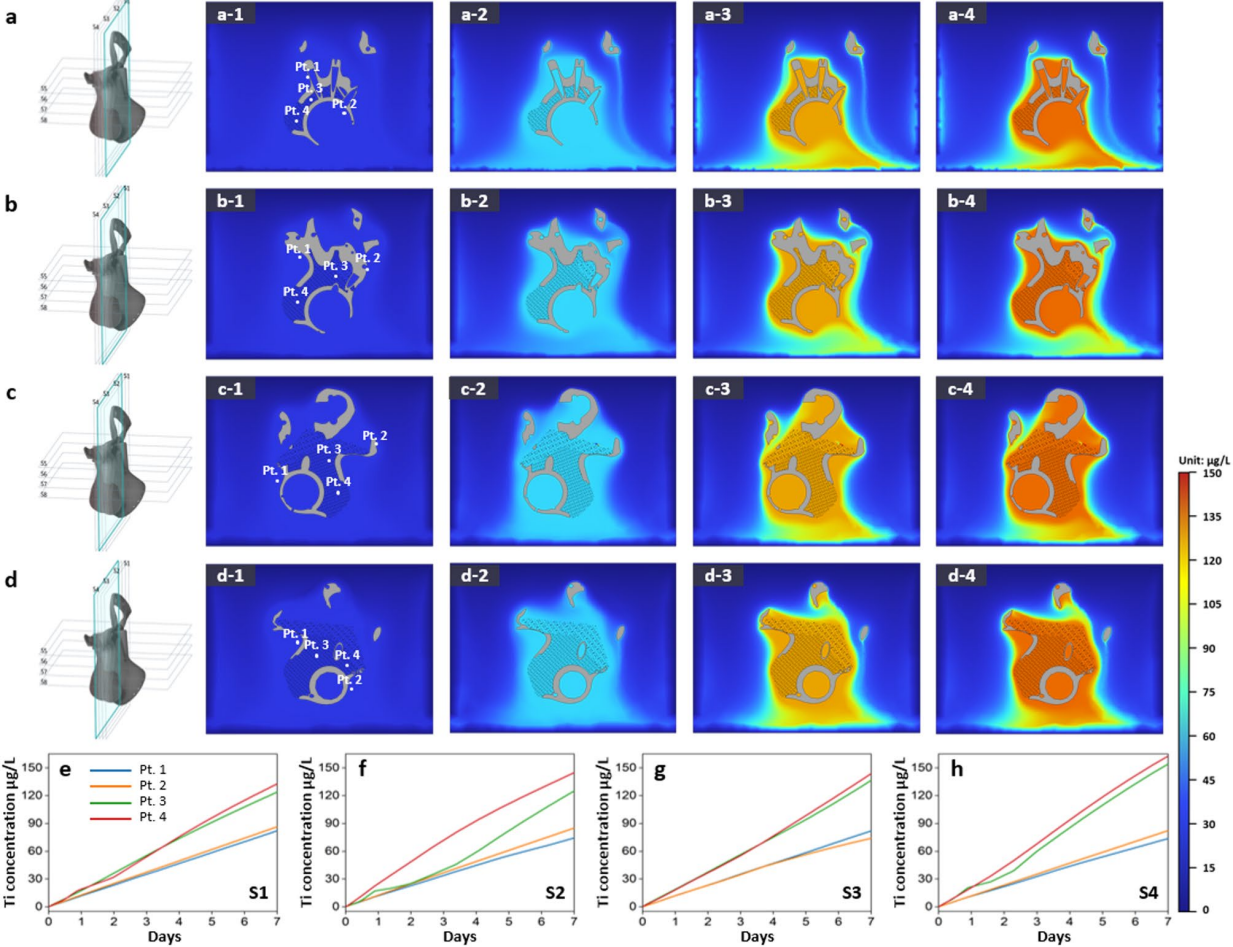
Ti dissolution behavior of a pelvic implant: simulation results showing the titanium dissolution at different cross-sections (S1 to S4) in Hartmann’s solution over 7 days. Panels (**a**) to (**d**) represent the dissolution behavior at each cross-section, where the 3D schematic on the left (modeled in SpaceClaim 2021 R1) indicates the exact slicing location. For each section, (1) shows the monitoring points (Pt. 1 to Pt. 4), with Pt. 1 and Pt. 2 located near the solid region and Pt. 3 and Pt. 4 located near the lattice structure. Panels (2)–(4) illustrate the distribution of dissolved Ti ions after 2, 4, and 7 days, respectively. Panels (**e**) to (**h**) display the Ti ion concentration trends over 7 days at the four monitoring points for cross-sections S1 to S4. The color gradient indicates the concentration of dissolved titanium ions (in µg/L). Dissolution simulation results were generated using COMSOL Multiphysics v6.0.

**Fig. 8:**
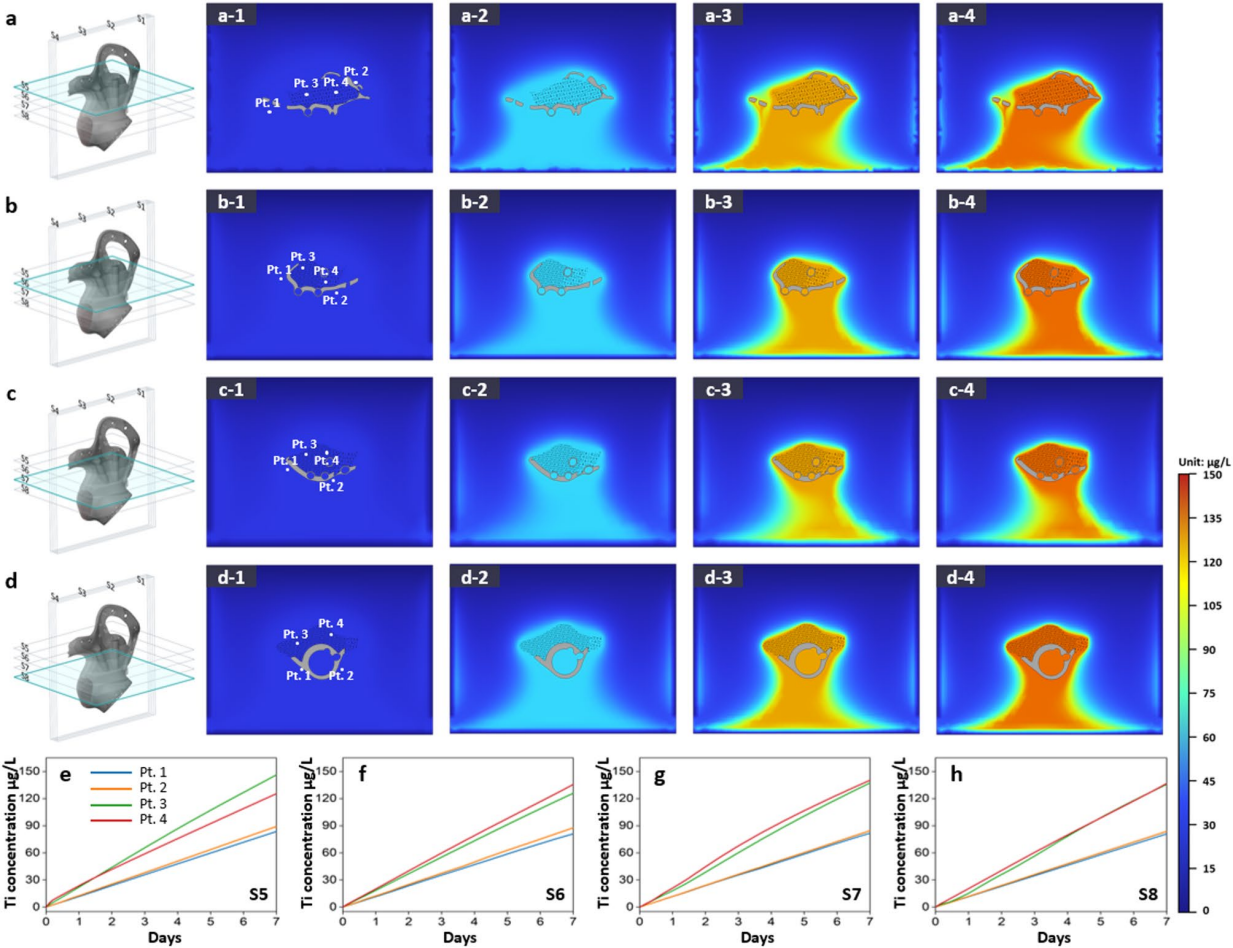
Ti dissolution behavior of a pelvic implant: simulation results through horizontal cross-sectional analysis. Simulation results showing titanium dissolution from pelvic implants at horizontal slices (S5 to S8) in Hartmann’s solution over 7 days. Panels (**a**) to (**d**) represent dissolution behavior at each slice, where the 3D schematic on the left indicates the corresponding slicing location. For each slice, (1) shows the four monitoring points (Pt. 1 to Pt. 4), and (2)–(4) illustrate the distribution of dissolved Ti ions after 2, 4, and 7 days, respectively. Panels (**e**) to (**h**) show the Ti ion concentration trends at the four monitoring points for slices S5 to S8. The color gradient indicates the concentration of dissolved titanium ions (in µg/L).

**Fig. 9:**
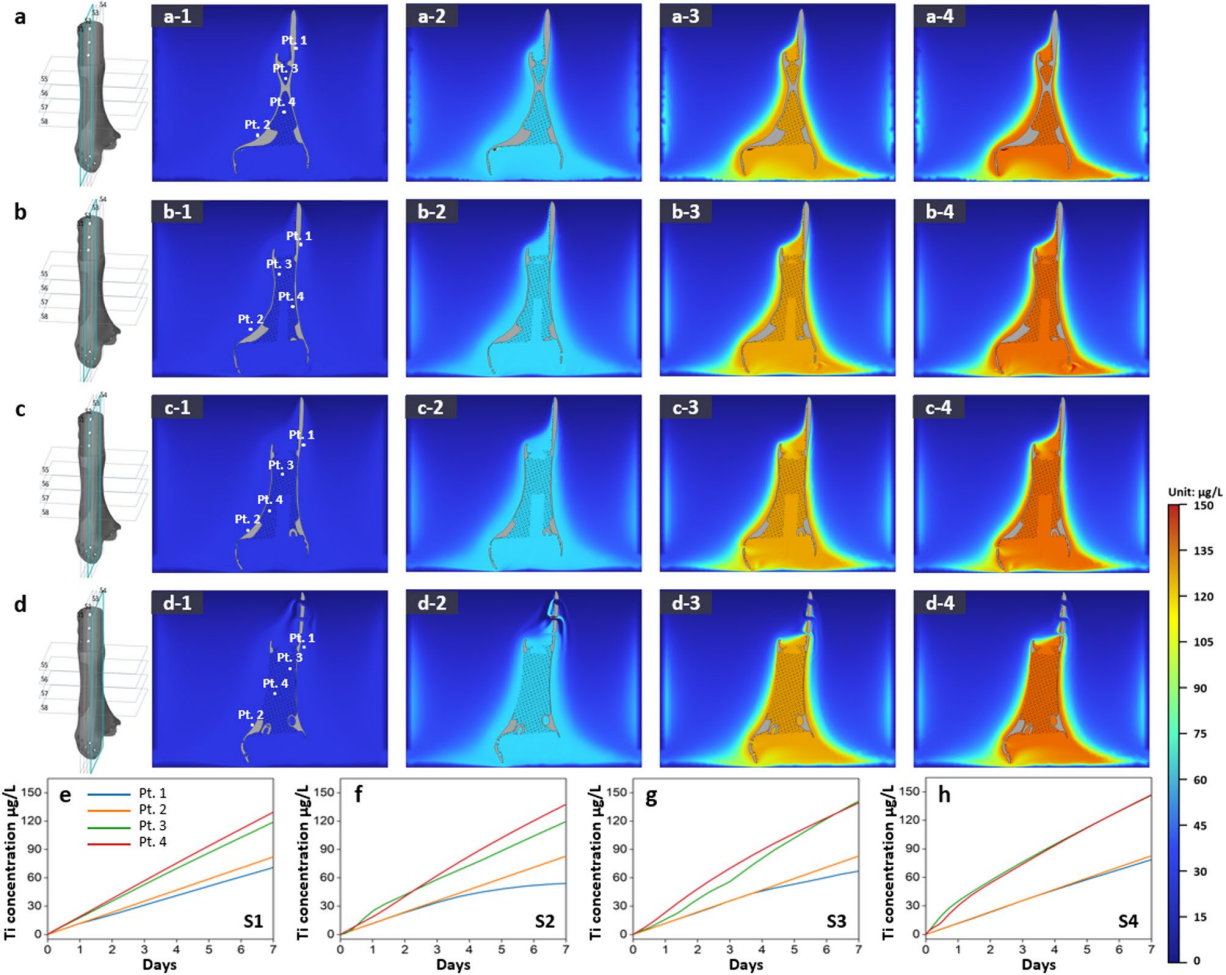
Ti dissolution behavior of a tibial implant: simulation results showing the titanium dissolution at different cross-sections (S1 to S4) in Hartmann’s solution over 7 days. Panels (**a**) to (**d**) represent the dissolution behavior at each cross-section, where the 3D schematic on the left indicates the exact slicing location. For each section, (1) shows the monitoring points (Pt. 1 to Pt. 4), with Pt. 1 and Pt. 2 located near the solid region and Pt. 3 and Pt. 4 located near the lattice structure. Panels (2)–(4) illustrate the distribution of dissolved Ti ions after 2, 4, and 7 days, respectively. Panels (**e**) to (**h**) display the Ti ion concentration trends over 7 days at the four monitoring points for cross-sections S1 to S4. The color gradient indicates the concentration of dissolved titanium ions (in µg/L).

**Fig. 10:**
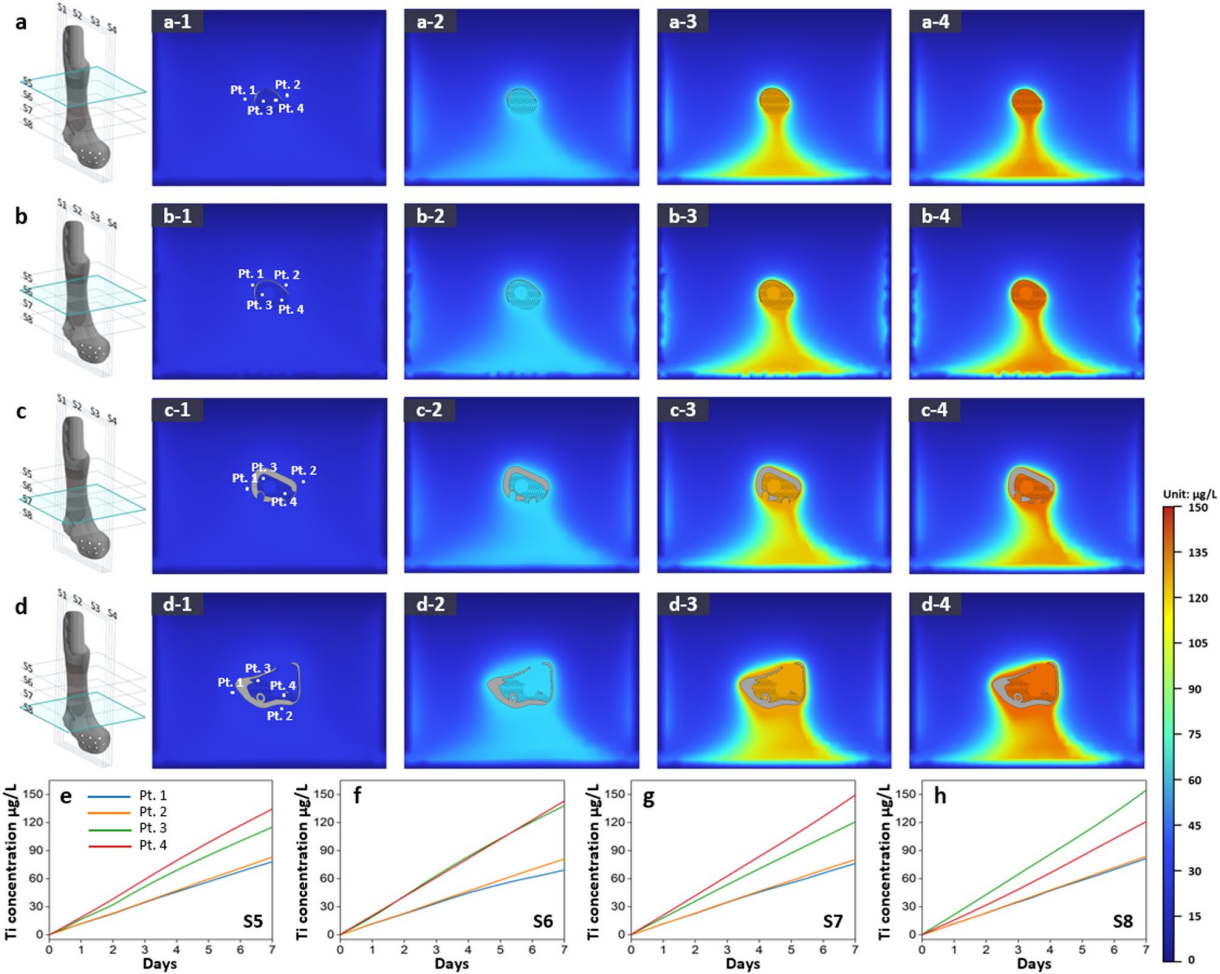
Ti dissolution behavior of a tibial implant: simulation results through horizontal cross-sectional analysis. Simulation results showing titanium dissolution from tibial implants at horizontal slices (S5 to S8) in Hartmann’s solution over 7 days. Panels (**a**) to (**d**) represent dissolution behavior at each slice, where the 3D schematic on the left indicates the corresponding slicing location. For each slice, (1) shows the monitoring points (Pt. 1 to Pt. 4), and (2)–(4) illustrate the distribution of dissolved Ti ions after 2, 4, and 7 days, respectively. Panels (**e**) to (**h**) display the Ti ion concentration trends at the four monitoring points for slices S5 to S8. The color gradient indicates the concentration of dissolved titanium ions (in µg/L).

Previous simulations investigated titanium specimens, whereas subsequent analyses focused on full pelvic and tibial implants to better evaluate dissolution behavior in clinically relevant scenarios. Figures 7 and 8 show the titanium dissolution behavior of a pelvic implant through vertical and horizontal cross-sectional analyses, respectively, in Hartmann’s solution over 7 days. Figures 9 and 10 present the corresponding results for a tibial implant. The analysis is based on 3D model representations, cross-sectional simulation results, and point-specific dissolution trends.

For the pelvic implant, the vertical cross-sectional analysis (Figs. 7a–d) reveals how dissolution behavior evolves across sections S1 to S4. Each schematic on the left clearly designates the slicing location. The sub-panels labeled (1) identify four monitoring points—Pt. 1 and Pt. 2 near the solid region, and Pt. 3 and Pt. 4 closer to the lattice structure. Panels (2) to (4) visualize Ti ion distribution at 2, 4, and 7 days. Time-course plots in Figs. 7e–h display how Ti ion levels changed at each point. Notably, at cross-section S3, the concentration at Pt. 4 after 7 days reached approximately 143.77 µg/L, whereas Pt. 1 showed only 81.94 µg/L, indicating nearly a 75% increase near the lattice zone. Moreover, across sections S1 to S4, the proportion of lattice structure within the cross-section progressively increases, with corresponding Ti ion concentrations rising from 132.68 µg/L in S1 to 162.77 µg/L in S4 at lattice-adjacent points. This trend suggests that a higher lattice volume fraction contributes to more pronounced Ti dissolution, likely due to the enlarged reactive surface area and increased exposure to the electrolyte environment.

In contrast, horizontal cross-sections of the same pelvic implant are presented in Fig. 8, spanning slices S5 to S8. The visualization format mirrors that of the vertical analysis, maintaining consistent placement of monitoring points. As time progresses, contour maps (Figs. 8a–d) depict the gradual expansion of Ti ion fields, while corresponding graphs (Figs. 8e–h) illustrate the temporal concentration changes. For instance, in slice S6, Pt. 3 exhibited a Ti ion concentration of 125.86 µg/L after 7 days, whereas Pt. 1 remained at 81.02 µg/L, again emphasizing the elevated dissolution rate adjacent to the lattice region.

To extend this analysis to another anatomical site, Figs. 9 and 10 present the vertical and horizontal cross-sectional simulation results for a tibial implant, using the same slicing scheme (S1 to S4 for vertical, S5 to S8 for horizontal). As with the pelvic implant, each slice includes distribution maps of dissolved Ti ions over time and line plots showing the Ti ion concentration trends at the four monitoring points. In both vertical (Fig. 9) and horizontal (Fig. 10) cross-sections of the tibial implant, Ti ion concentrations at Pt. 3 and Pt. 4—located near the lattice region—showed a steeper increase over time and reached higher concentrations than those at Pt. 1 and Pt. 2, which are positioned near the solid structure. For instance, in vertical slice S2, the Ti concentration at Pt. 4 after 7 days reached approximately 137.74 µg/L, whereas Pt. 2 showed only 82.92 µg/L. A similar trend was evident in horizontal slice S7, where Pt. 3 recorded 138.04 µg/L compared to 81.15 µg/L at Pt. 2. These data reaffirm that dissolution behavior is significantly intensified in regions adjacent to lattice structures, likely due to their larger surface area and microstructural porosity.

In the simulation results presented in Figs. 7, 8, 9 and 10 and a slight downward trend in Ti ion concentration was observed. While gravitational forces can, in theory, cause ion sedimentation, the diffusion coefficient of Ti⁴⁺ in aqueous solutions (~ 1.1 × 10⁻⁶ cm²/s) indicates that diffusion dominates over sedimentation under the experimental conditions and timescales considered. Therefore, the observed ion distribution is primarily attributed to diffusion-driven processes rather than gravitational settling^47^.

The non-uniform distribution of Ti ions in the simulated results can be attributed to both structural and microstructural heterogeneity. Lattice regions provide a significantly larger exposed surface area, leading to greater localized dissolution, while the galvanic coupling between α and β phases further accelerates corrosion in specific regions. As a result, these effects generate spatial gradients in Ti ion concentration around the specimens.

Across all cases, the simulation results reveal that the dissolution behavior and Ti ion elution are strongly influenced by both the lattice distribution and the anatomical geometry. Monitoring point analyses consistently showed higher Ti ion concentrations near lattice regions (Pt. 3 and Pt. 4) than near solid regions (Pt. 1 and Pt. 2), regardless of slicing direction or anatomical site. Higher lattice volume fractions led to greater Ti ion release, while the spatial pattern of dissolution varied depending on implant geometry and the orientation of the cross-sectional slice. These findings emphasize the importance of lattice design optimization in 3D-printed implants to balance mechanical stability and corrosion resistance.

**Fig. 11:**
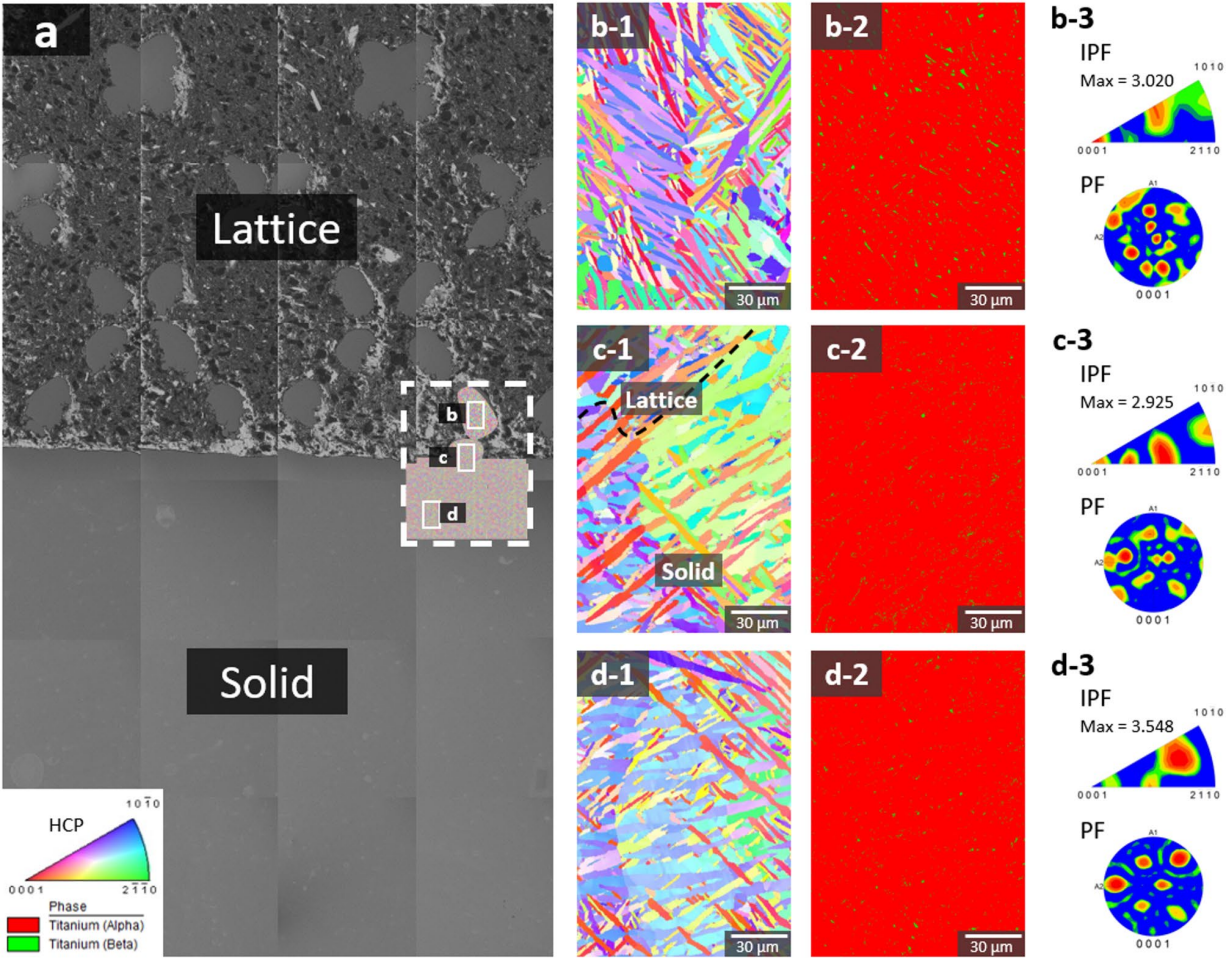
Electron backscatter diffraction (EBSD) analysis of hybrid-structure specimens subjected to dissolution testing: (**a**) specimen fabricated by L-PBF. (**b-1**, **c-1**, **d-1**) Inverse pole figure map, (**b-2**, **c-2**, **d-2**) phase map, texture analysis results of (**b-3**) lattice, (**c-3**), boundary, and (**d-3**) solid regions for the L-PBF specimen.

Microstructural behavior after dissolution was analyzed for solid area, solid-lattice interface area, and lattice area with hybrid specimen as shown in Fig. 11. The specimen fabricated by L-PBF exhibits distinct lattice and solid regions, with the lattice region displaying a porous, interconnected structure, while the solid region is denser and more uniform. The inverse pole figure (IPF) maps of the lattice, boundary, and solid regions (Fig. 11b-1, c-1, d-1 highlight the crystallographic orientation of the microstructures. The lattice region exhibits a diverse grain orientation due to the complex thermal gradients and rapid cooling rates during L-PBF processing, whereas the solid region shows a more homogeneous texture, indicative of consistent solidification conditions.

Phase maps corresponding to the lattice, boundary, and solid regions (Fig. 11b-2, c-2, d-2) distinguish between the α phase and β phase, where the α phase is depicted in red. The results demonstrate a higher prevalence of the β phase within the lattice and boundary regions, while the solid region primarily exhibits the α phase structure. This phase distribution suggests that the lattice regions contain a higher fraction of retained β phase due to the rapid cooling rates associated with L-PBF processing. The finer microstructure and increased surface area in the lattice regions promote higher cooling rates, which hinder the complete transformation of β phase to α phase, resulting in a greater retention of metastable β phase.

Texture analysis results (Figs. 11b-3, c-3, d-3) provide quantitative measurements of crystallographic texture through IPF and pole figure (PF) analyses. The crystallographic texture analysis reveals that the solid structure exhibits a stronger texture compared to the lattice structure. This difference indicates that the solid structure possesses a more pronounced alignment of grains, likely resulting from the uniform solidification conditions during L-PBF processing.

**Fig. 12:**
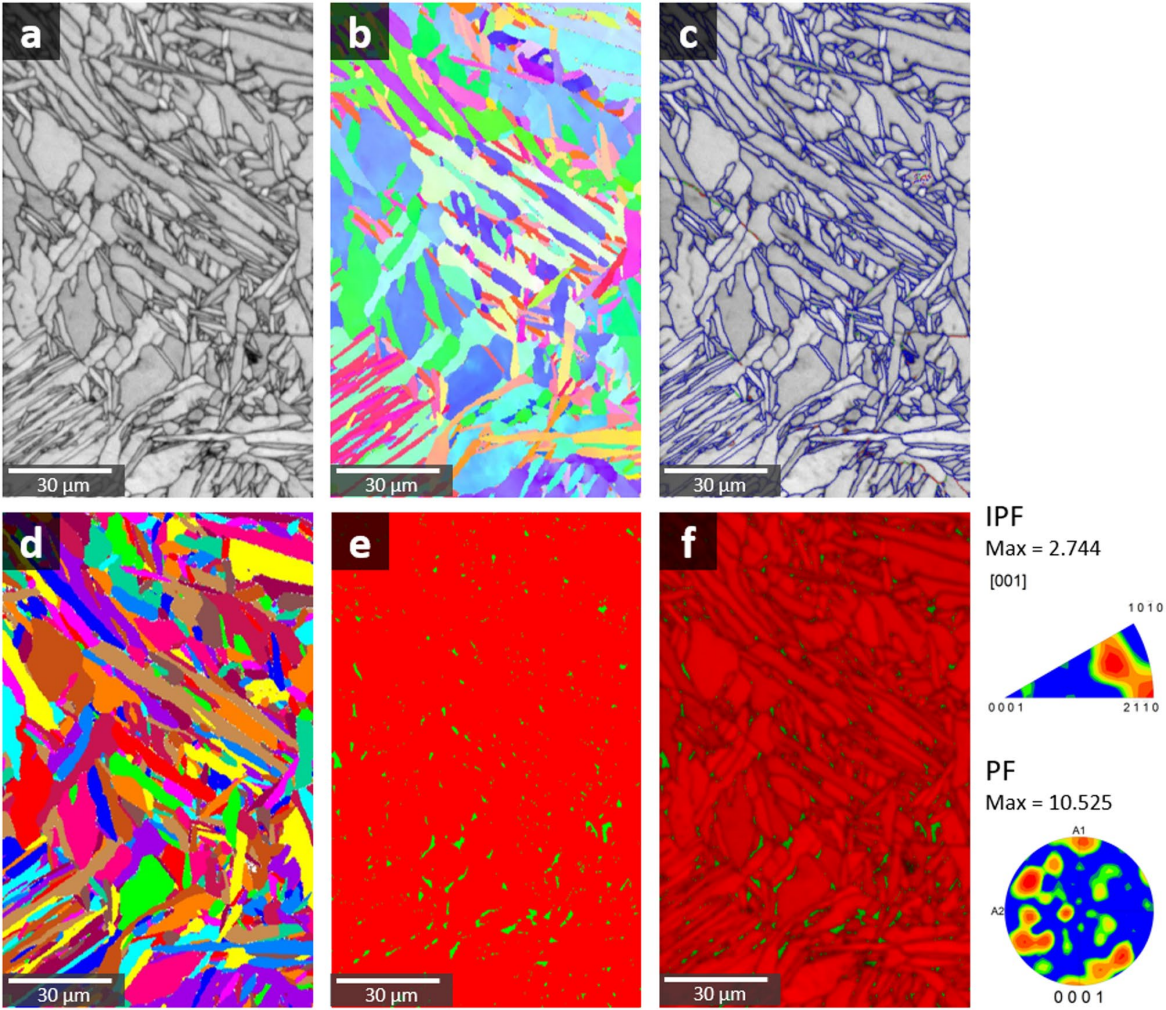
EBSD analysis of lattice-structure specimens subjected to dissolution testing: (**a**) Image quality map (**b**) Inverse pole figure map (**c**) Grain boundary map (**d**) Grain map (**e**) Phase map (**f**) Image quality + phase map.

Figure 12 presents a focused analysis of the lattice-structure specimens under dissolution testing conditions using EBSD (Electron backscatter diffraction). The image quality map (Fig. 12a) shows the overall microstructural morphology, highlighting the fine, interconnected microstructure typical of lattice-structured Ti6Al4V fabricated through LPBF. The elongated and irregular grain shapes indicate the influence of rapid solidification and thermal gradients during the additive manufacturing process.

The IPF map (Fig. 12b) represents the crystallographic orientation of the grains. The map reveals a heterogeneous distribution of crystal orientations, typical of LPBF specimens where localized thermal fluctuations during processing induce varied crystallographic textures. The IPF maximum intensity is 2.744, indicating moderate texture within the lattice structure. Throughout all regions, prior β grains grew along the 〈100〉 direction, followed by the formation of α laths within the prior β grains. All regions exhibited nearly isotropic properties owing to their weaker texture, an outcome typical of L-PBF processes^55^.

The grain boundary map (Fig. 12c) visualizes the grain boundaries within the lattice structure. The grain boundary map shows that the structure is primarily composed of high-angle grain boundaries (above 15°), with sub-structures not being well-developed. The grain map (Fig. 12d) illustrates the spatial distribution of individual grains within the lattice. The map confirms the presence of a heterogeneous grain structure with varying grain sizes and orientations, consistent with the thermal gradients and rapid solidification inherent to L-PBF processes.

The phase map (Fig. 12e) distinctly differentiates between the α phase, shown in red, and the β phase, shown in green. The high cooling rate in the lattice and hybrid regions promotes the retention of a relatively higher amount of β phase compared to the solid structure. Quantitatively, β phase percentages were measured as follows: 1.1% in the solid region, 1.6% in the hybrid region, and 2.2% in the lattice region. These values were obtained from EBSD analysis performed at relatively low magnification (x1200), which allowed a broad field of view to cover a representative area of each region. This approach minimizes local bias and provides reliable phase fraction data for structural comparison. This higher β phase fraction in the lattice region reflects the rapid cooling conditions during L-PBF processing, which suppresses the complete transformation of β phase to α phase.

The combined image quality and phase map (Fig. 12f) provide a visual correlation between microstructural quality and phase distribution. The green β phase is more prominent in regions with fine microstructural features, while the red α phase predominantly appears in more massive grain areas. This combination highlights the intricate relationship between microstructural heterogeneity and phase transformation tendencies in the lattice structure.

The strong texture in IPF and PF indicates a preferred crystallographic orientation within the lattice structure. These microstructural behaviors indicate the formation of a fine galvanic cell between the α and β phases, which leads to corrosion of the implant and a more active corrosion manner in the lattice area. The higher β phase fraction in the lattice region suggests a greater susceptibility to galvanic corrosion, potentially compromising the mechanical stability and corrosion resistance of the implant.

## Discussion

The lattice structures in 3D-printed implants are crucial for promoting bone and soft tissue ingrowth and reducing stress shielding^31,56^. Such lattice structure provides a porous framework that facilitates cellular infiltration and vascularization, fostering a conducive environment for tissue integration. However, the large surface area of lattice structures raises concerns regarding accelerated corrosion. Specifically, the large surface area, while advantageous for cellular interactions, may render the implant susceptible to corrosive processes. Consequently, the utilization of lattice structures necessitates a judicious approach, incorporating a comprehensive risk–benefit assessment. Striking a balance between the advantageous biological integration and potential corrosion risks is imperative in the selection and application of lattice structures within 3D-printed implants to ensure optimal performance and long-term biocompatibility.

In terms of metal ion toxicity within the human body, Ti is generally regarded as safe. Nevertheless, concerns arise owing to the presence of Ti in the bloodstream resulting from corrosion, primarily in the form of TiO_2_. The potential toxicity of TiO_2_ remains a subject of debate within the scientific community, introducing a level of complexity to its perceived safety^57^. Furthermore, Ti6Al4V, which is commonly used in orthopedic applications, may contain Al and V, both known to possess toxic properties^48,49^. Therefore, prudent consideration and caution are warranted when assessing the biocompatibility of Ti-based materials, taking into account the alloy components.

If the pores or defects in a 3D-printed implant fail to regenerate the TiO_2_ layer as corrosion progresses in the biological environment, a corrosive environment may be created, which may persist for several months or up to a year^28^. Such environments may lead to the generation of various defects, such as pitting, crevices, and cracks on the implant surface, which may deteriorate the mechanical properties of the implant^14^. Moreover, orthopedic implants are subjected to cyclical loads, such as during walking or running, which may induce stress concentrations in defective areas. This stress accumulation may accelerate the initiation of cracks, negatively impacting the fatigue life of the implant and reducing its fatigue strength^16^.

The 3D printing process introduces pores and defects during the fabrication process. By optimizing key printing parameters, including laser power, scan velocity, and layer thickness^17^, corrosion can be mitigated through the minimization of pitting or defects in environments lacking a sufficient oxide layer. After fabrication, heat treatment can be applied to further minimize pores and defects. Finally, a sufficient amount of oxide layer can be created through post-processing techniques, such as anodizing, and the corrosion resistance can be further enhanced by developing a nanotube oxide layer on the surface^58,59^.

This study has several limitations that should be acknowledged. While our experiments and simulations focused on metal ion dissolution, we did not evaluate the biological consequences of released ions. Future studies should investigate the effects of Ti, Al, and V ions on orthopedic-relevant cell types (e.g., osteoblasts and mesenchymal stem cells) in vitro and in vivo to clarify how ion release may influence bone regeneration, osseointegration, and long-term implant stability. In addition, although the comparison between ultrapure water and Hartmann’s solution provided indirect insight into particle wash-out versus corrosion-driven release, our design did not fully differentiate these mechanisms. Direct morphological analyses of soaking solutions (e.g., SEM or nanoparticle tracking) would be valuable to clarify the nature of released debris. Electrochemical techniques such as potentiodynamic polarization and electrochemical impedance spectroscopy, which are widely used to evaluate corrosion kinetics and passive film stability, were beyond the scope of this work but could provide complementary insights when integrated with long-term dissolution testing. Despite these limitations, the present study offers important evidence linking implant structure and microstructure to ion dissolution behavior, providing a foundation for future efforts to optimize 3D-printed orthopedic implant design.

## Conclusion

The blood Ti concentration after orthopedic surgery using a 3D printed implant was identified through the dissolution experiments and simulation of solid, hybrid and lattice specimens. A comparative analysis of FEA results for 3D-printed specimens shows that the hybrid eluted 111% more Ti ions, and the lattice eluted 130% more Ti ions in ultrapure water, based on solid specimens. In Hartmann’s solution, hybrid eluted 109% and lattice eluted 154% more Ti ions based on solid specimen. In dissolution experiments on 7 d after wash-out results were compared based on MUTARS, in ultrapure water, solid recorded 14%, hybrid recorded 29%, and lattice recorded 29% Ti ion concentration. However, in Hartman’s solution, concentrations of 180% for solid, 339% for hybrid, and 464% for lattice were analyzed, and as the specific surface area increased, the Ti ion concentration was higher. In EBSD analysis, although the variances in grain boundary lengths were subtle in each region, there was a notable difference in the β phase content, ranging from 145% to 200%. The higher β phase content in the lattice region can be attributed to its higher Ti dissolution. Titanium is generally known for its low toxicity; however, in cases where sustained corrosion of the implant occurs within the body, there may be potential risks associated with elevated metal concentrations. Moreover, apart from toxicity issues arising from high metal concentrations, mechanisms such as the expansion of defects within 3D-printed implants due to the progression of corrosion can render them more vulnerable to fatigue failure. Reflecting the findings of this study, 3D-printed artificial implant durability can be affected by the dissolution effect associated with the different mesh-solid hybrid design. Further studies may be done to find optimal 3D-printed implant design considering statical, dynamical and chemical aspect all together.

## Data Availability

The datasets analyzed during the current study are not publicly available due to privacy restrictions, but are available from the corresponding author on reasonable request. Requests for data should be directed to Prof. Im Doo Jung ( [idjung@unist.ac.kr](mailto: idjung@unist.ac.kr) ).

## References

[1] Park, S., Kim, J. G., Jung, I. D., Seol, J. B. & Sung, H. Ultrastrong and stress corrosion cracking-resistant martensitic steels. *Acta Mater*. **239**, 118291 (2022).

[2] Joshi, S. C. & Sheikh, A. A. 3D printing in aerospace and its long-term sustainability. *Virtual Phys. Prototyp.* **10**, 175–185 (2015).

[3] Prashar, G., Vasudev, H. & Bhuddhi, D. Additive manufacturing: expanding 3D printing horizon in industry 4.0. *Int. J. Interact. Des. Manuf.* **17**, 2221–2235 (2023). 10.1007/s12008-022-00956-4 PMC9256535 40479213

[4] Kumar, A., Mishra, D. K., Upadhyay, L., Srivastava, M. & Pathak, D. K. Additive manufacturing technologies for on-demand production of personalized goods. In *Laser Applications in Manufacturing*. 120–152 (CRC Press, 2023).

[5] Jung, I. D. et al. Embedding sensors using selective laser melting for self-cognitive metal parts. *Addit. Manuf.* **33** (2020).

[6] Ashima, R., Haleem, A., Javaid, M. & Rab, S. Understanding the role and capabilities of internet of things-enabled additive manufacturing through its application areas. In *Advanced Industrial and Engineering Polymer Research*. Vol. 5. 137–142. Preprint (2022). 10.1016/j.aiepr.2021.12.001

[7] Panesar, A., Abdi, M., Hickman, D. & Ashcroft, I. Strategies for functionally graded lattice structures derived using topology optimisation for additive manufacturing. *Addit. Manuf.* **19**, 81–94 (2018).

[8] Aimar, A., Palermo, A. & Innocenti, B. The role of 3D printing in medical applications: A state of the art. *J. Healthc. Eng.* **2019**. Preprint (2019). 10.1155/2019/5340616 10.1155/2019/5340616 PMC6451800 31019667

[9] Lee, J. et al. Microstructural effects on the tensile and fracture behavior of selective laser melted H13 tool steel under varying conditions. *Mater. Charact*. **155** (2019).

[10] Zadeh, M. K., Yeganeh, M., Shoushtari, M. T., Ramezanalizadeh, H. & Seidi, F. Microstructure, corrosion behavior, and biocompatibility of Ti-6Al-4 V alloy fabricated by LPBF and EBM techniques. *Mater. Today Commun.* **31** (2022).

[11] Martin, É., Azzi, M., Salishchev, G. A. & Szpunar, J. Influence of microstructure and texture on the corrosion and tribocorrosion behavior of Ti-6Al-4V. *Tribol Int.* **43**, 918–924 (2010).

[12] Lee, M. S. et al. Selective laser melting process for sensor embedding into SUS316L with heat dissipative inner cavity design. *Met. Mater. Int.* **28**, 297–305 (2022).

[13] Rodrigues, D. C., Urban, R. M., Jacobs, J. J. & Gilbert, J. L. In vivo severe corrosion and hydrogen embrittlement of retrieved modular body titanium alloy hip-implants. *J. Biomed. Mater. Res. B Appl. Biomater.* **88**, 206–219 (2009). 18683224 10.1002/jbm.b.31171 PMC2667129

[14] Cui, Y. W., Chen, L. Y. & Liu, X. X. Pitting corrosion of biomedical titanium and titanium alloys: A brief review. *Curr. Nanosci.* **17**, 241–256 (2021).

[15] Seo, E. et al. Laser powder bed fusion for AI assisted digital metal components. *Virtual Phys. Prototyp.* **17**, 806–820 (2022).

[16] de Jesus, J., Ferreira, J. A. M., Borrego, L., Costa, J. D. & Capela, C. Fatigue failure from inner surfaces of additive manufactured ti-6al-4v components. *Materials* **14**, 1–12 (2021). 10.3390/ma14040737 PMC7915358 33562437

[17] Mojumder, S. et al. Linking process parameters with lack-of-fusion porosity for laser powder bed fusion metal additive manufacturing. *Addit. Manuf.* **68** (2023).

[18] Kim, T. et al. Virtual surface morphology generation of Ti-6Al-4V directed energy deposition via conditional generative adversarial network. *Virtual Phys. Prototyp.* **18** (2023).

[19] Gilday, D. L. & Ash, J. M. *Benign Bone Tumors*. 10.1016/s0001-2998(76)80034-7 1082170

[20] Woertler, K. Benign bone tumors and tumor-like lesions: Value of cross-sectional imaging. Eur. Radiol. **13**, 1820–1835. Preprint (2003). 10.1007/s00330-003-1902-z 10.1007/s00330-003-1902-z 12700923

[21] Rigor, B. M. Pelvic cancer pain. *J. Surg. Oncol.* **75**, 280–300. Preprint (2000). 10.1002/1096-9098(200012)75:4%3C280::AID-JSO13%3E3.0.CO;2-Q 10.1002/1096-9098(200012)75:4<280::aid-jso13>3.0.co;2-q 11135274

[22] Hillmann, A. et al. Tumors of the pelvis: complications after reconstruction. *Arch. Orthop. Trauma. Surg.* **123**, 340–344 (2003). 12838435 10.1007/s00402-003-0543-7

[23] Liang, H., Ji, T., Zhang, Y., Wang, Y. & Guo, W. Reconstruction with 3D-printed pelvic endoprostheses after resection of a pelvic tumour. *Bone Joint J.* **99**, 267–275 (2017). 28148672 10.1302/0301-620X.99B2.BJJ-2016-0654.R1

[24] Buj-Corral, I., Tejo-Otero, A. & Fenollosa-Artés, F. Development of am technologies for metals in the sector of medical implants. *Metals* **10** Preprint (2020). 10.3390/met10050686

[25] Park, J. W. et al. In vivo analysis of post-joint-preserving surgery fracture of 3D-printed Ti-6Al-4V implant to treat bone cancer. *Biodes Manuf.* **4**, 879–888 (2021).

[26] Park, J. W. et al. Hybrid solid mesh structure for electron beam melting customized implant to treat bone cancer. *Int. J. Bioprint*. **9**, 1–14 (2023). 10.18063/ijb.716 PMC10261134 37323484

[27] Lee, J. et al. Reverse effect of hot isostatic pressing on high-speed selective laser melted Ti–6Al–4V alloy. *Mater. Sci. Eng. A* **807**, 140880 (2021).

[28] Balasubramanian Gayathri, Y. K. et al. Additive manufacturing of Ti-6Al-4V alloy for biomedical applications. *J. Bio Tribocorros.* **8** (2022).

[29] Jardini, A. L. et al. Customised titanium implant fabricated in additive manufacturing for craniomaxillofacial surgery: this paper discusses the design and fabrication of a metallic implant for the reconstruction of a large cranial defect. *Virtual Phys. Prototyp.* **9**, 115–125 (2014).

[30] Angelini, A. et al. Analysis of principles inspiring design of three-dimensional-printed custom-made prostheses in two referral centres. *Int. Orthop.* **44**, 829–837 (2020). 32170471 10.1007/s00264-020-04523-y

[31] Park, J. W., Kang, H. G., Kim, J. H. & Kim, H. S. New 3-dimensional implant application as an alternative to allograft in limb salvage surgery: a technical note on 10 cases. *Acta Orthop.* **91**, 489–496 (2020). 32396448 10.1080/17453674.2020.1755543 PMC8023892

[32] Park, J. W., Kang, H. G., Kim, J. H. & Kim, H. S. The application of 3D-printing technology in pelvic bone tumor surgery. *J. Orthop. Sci.* **26**, 276–283 (2021). 32247647 10.1016/j.jos.2020.03.004

[33] Kang, H. G. *Clinical Atlas of 3D Printing Bone Reconstruction* (Springer, 2021).

[34] Long, M. & Rack, H. J. Titanium alloys in total joint replacement-A materials science perspective. *Biomaterials* **19** (1998). 10.1016/s0142-9612(97)00146-4 9839998

[35] Liu, X., Chu, P. K. & Ding, C. Surface modification of titanium, titanium alloys, and related materials for biomedical applications. *Mater. Sci. Eng. R Rep*. **47**, 49–121. Preprint (2004). 10.1016/j.mser.2004.11.001

[36] Geetha, M., Singh, A. K., Asokamani, R. & Gogia, A. K. Ti based biomaterials, the ultimate choice for orthopaedic implants - A review. *Prog. Mater. Sci*. **54**, 397–425. Preprint (2009). 10.1016/j.pmatsci.2008.06.004

[37] Swiatkowska, I., Martin, N. & Hart, A. J. Blood titanium level as a biomarker of orthopaedic implant wear. *J. Trace Elem. Med. Biol*. **53**, 120–128. Preprint (2019). 10.1016/j.jtemb.2019.02.013 10.1016/j.jtemb.2019.02.013 30910194

[38] Kaur, M. & Singh, K. Review on titanium and titanium based alloys as biomaterials for orthopaedic applications. *Mater. Sci. Eng. C* **102**, 844–862. Preprint (2019). 10.1016/j.msec.2019.04.064 10.1016/j.msec.2019.04.064 31147056

[39] Tigani, D., Fosco, M., Ayad, R., Ben & Fantasia, R. Orthopaedic implant materials and design. In *Wear of Orthopaedic Implants and Artificial Joints*. 133–177 (Elsevier, 2013).

[40] Ask, M., Lausmaa, J. & Kasemo, B. Preparation and surface spectroscopic characterization of oxide films on Ti6A14V. *Appl. Surf. Sci.* **35**, 283–301 (1989).

[41] Katti, K. S., Verma, D. & Katti, D. R. . Materials for joint replacement. In *Joint Replacement Technology*. 81–104 (Elsevier, 2008).

[42] Omlor, G. W. et al. In vivo serum titanium ion levels following modular neck total hip arthroplasty-10 year results in 67 patients. *Acta Biomater.* **9**, 6278–6282 (2013). 23232209 10.1016/j.actbio.2012.12.001

[43] Grosse, S. et al. Wear particles and ions from cemented and uncemented titanium-based hip prostheses - A histological and chemical analysis of retrieval material. *J. Biomed. Mater. Res. B Appl. Biomater.* **103**, 709–717 (2015). 25051953 10.1002/jbm.b.33243 PMC4413358

[44] Leopold, S. S. et al. Serum titanium level for diagnosis of a failed, metal-backed patellar component. *J. Arthroplasty*. **15**, 938–943 (2000). 11061457 10.1054/arth.2000.6632

[45] Takai, S., Yoshino, N., Kusaka, Y., Watanabe, Y. & Hirasawa, Y. Dissemination of metals from a failed patellar component made of titanium-base alloy. *J. Arthroplasty* **18** (2003). 10.1016/s0883-5403(03)00277-8 14566752

[46] Hallab, N. J. et al. Effects of soluble metals on human peri-implant cells. *J. Biomed. Mater. Res. A*. **74**, 124–140 (2005). 15937919 10.1002/jbm.a.30345

[47] Okazaki, Y., Rao, S., Ito, Y. & Tateishi, T. C. Resistance mechanical properties, corrosion fatigue strength and cytocompatibility of new Ti alloys without Al and V. *Biomaterials* **19** (1998). 10.1016/s0142-9612(97)00235-4 9720903

[48] Klein, G. L. Aluminum toxicity to bone: A multisystem effect? *Osteoporos. Sarcopenia*. **5**, 2–5 (2019). 31008371 10.1016/j.afos.2019.01.001 PMC6453153

[49] Ścibior, A., Pietrzyk, Ł., Plewa, Z. & Skiba, A. Vanadium Risks and possible benefits in the light of a comprehensive overview of its pharmacotoxicological mechanisms and multi-applications with a summary of further research trends.* J. Trace Elem. Med. Biol.* **61**. Preprint (2020). 10.1016/j.jtemb.2020.126508 10.1016/j.jtemb.2020.126508 PMC7152879 32305626

[50] Sharma, P., Pathak, D. K. & Gowerneni, A. Simulation model for the corrosion behavior of additively manufactured iron in electrolytic environment using COMSOL multi-physics. *NanoWorld J.* **9**, S260–S265 (2023).

[51] Gupta, D., Kumar, Y., Prajapati, V., Kalam, A. & Dubey, M. Time dependent analysis of galvanic corrosion on mild steel with magnesium alloy (AE44) Rivet-plate joint system using COMSOL multiphysics simulation. *J. Bio Tribocorros.* **8** (2022).

[52] Chen, W. et al. Study on the preparation and corrosion resistance properties of superhydrophobic coatings on galvanized steel. *Metals (Basel)* **13** (2023).

[53] Ude, C. C. et al. The mechanism of metallosis after total hip arthroplasty. *Regener. Eng. Transl. Med*. **7**, 247–261. Preprint (2021). 10.1007/s40883-021-00222-1 10.1007/s40883-021-00222-1 PMC9075182 35530571

[54] Bayat, M. et al. Keyhole-induced porosities in laser-based powder bed fusion (L-PBF) of Ti6Al4V: High-fidelity modelling and experimental validation. *Addit. Manuf.* **30** (2019).

[55] Niendorf, T., Brenne, F. & Schaper, M. Lattice structures manufactured by SLM: on the effect of geometrical dimensions on microstructure evolution during processing. *Metall. Mater. Trans. B*. **45**, 1181–1185 (2014).

[56] Koju, N., Niraula, S. & Fotovvati, B. Additively manufactured porous Ti6Al4V for bone implants: A review. *Metals* **12**. Preprint (2022). 10.3390/met12040687

[57] Kim, K. T., Eo, M. Y., Nguyen, T. T. H. & Kim, S. M. General review of titanium toxicity. *Int J. Implant Dent.* **5** (2019). 10.1186/s40729-019-0162-x PMC6409289 30854575

[58] Durdu, S., Cihan, G., Yalcin, E. & Altinkok, A. Characterization and mechanical properties of TiO2 nanotubes formed on titanium by anodic oxidation. *Ceram. Int.* **47**, 10972–10979 (2021).

[59] Bocchetta, P. et al. Passive layers and corrosion resistance of biomedical Ti-6Al-4V and β-Ti alloys. *Coatings* **11**. Preprint (2021). 10.3390/coatings11050487

